# Synthesis, structure, and biological activity of novel heterocyclic sulfonyl-carboximidamides

**DOI:** 10.1007/s00706-012-0888-0

**Published:** 2013-01-25

**Authors:** Katarzyna Gobis, Henryk Foks, Jarosław Sławiński, Artur Sikorski, Damian Trzybiński, Ewa Augustynowicz-Kopeć, Agnieszka Napiórkowska, Krzysztof Bojanowski

**Affiliations:** 1 Department of Organic Chemistry, Medical University of Gdańsk, Gdańsk, Poland; 2 Department of Physical Chemistry, University of Gdańsk, Gdańsk, Poland; 3 Department of Microbiology, Institute of Tuberculosis and Pulmonary Diseases, Warsaw, Poland; 4 Sunny BioDiscovery, Santa Paula, CA USA

**Keywords:** Sulfonamidine, Heterocycles, Crystal structure, Antimicrobial activity, Antitumor activity, Structure–activity relationship

## Abstract

**Abstract:**

A series of novel heterocyclic sulfonyl-carboximidamides were synthesized in satisfactory yields via condensation of heterocyclic methyl carbimidates with 2-chlorobenzenesulfonamide and 4-chloropyridine-3-sulfonamide. New structures were confirmed by IR and NMR spectra as well as elemental analyses. X-ray crystallography of two derivatives was performed. The single-crystal structures confirmed the presence of a primary amine group in the amidine moiety. All the compounds were screened for their tuberculostatic, antibacterial, and anticancer activities. Preliminary results indicated that target compounds exhibited weak tuberculostatic and antibacterial activities. Seven compounds inhibited the growth of some cancer cell lines, whereas one of the 2-quinoline derivatives displayed favorable activity against all tested cancer cells with *GI*_50_ values of 0.92–13 μM.

**Graphical abstract:**

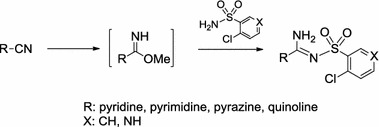

## Introduction

Sulfonamides are compounds with diverse pharmacological activity. They are known the most for their antibacterial [1] and antihypoglycemic [2] activities. Some of the sulfonamides act as antimycobacterial [3] and antifungal [4] agents. Intensive studies on the antitumor activity of sulfonamides were also carried out [5]. One of the most potent compounds is chloroquinoxaline sulfonamide (CQS) presently in the second phase of clinical trials [6, 7]. This compound has a chlorine atom in its structure linked to the quinoxaline ring (C-5) substituted at the C-2 position with a sulfanilamide moiety. The antitumor activity of this sulfonamide is associated with the inhibition of topoisomerase II [8]. Other sulfonamides have also been described as apoptosis promoters [9, 10] including sulfonyl-carboximidamides [11].

The amidine functional group is an important structural element of compounds with established pharmacological activity. Amidine derivatives have antidegenerative [12], antitumor [13], and antiplatelet action [14]. They also act as serine protease inhibitors [15] and nitric oxide synthase (NOS) inhibitors [16]. Compounds with anti-HIV [17], antibacterial, and antifungal activities [18] also were found among them. Moreover, the amidine group may be a perfect linker unit that could connect two pharmacophores, e.g., the sulfanilamide moiety and the pyridine or pyrazine system.

These findings prompted us to extend our search for biologically active compounds among nitrogen heterocyclic derivatives. We undertook the synthesis of structures that were condensates of heterocyclic methyl carbimidates with 2-chlorobenzenesulfonamide and 4-chloropyridine-3-sulfonamide. The synthesized compounds were evaluated for their biological activity in vitro: tuberculostatic, antibacterial, cytotoxic, and anticancer. We have also determined their crystal structure.

## Results and discussion

The subject of this work was to study the reactions of heterocyclic carbimidates with sulfonamides that have a chlorine atom as a substituent in the *ortho* position to the sulfonamide group. Carbimidates are compounds of great reactivity. Among others they react with amines giving amidines as the products. A few reactions of alkyl- and phenylcarbimidates with sulfonamides have been also described. As a result, sulfonamidines are formed [19].

One method presented in this article is to use 2-, 3-, and 4-pyridine-, 2-pyrimidine-, 2-pyrazine-, 6-chloro-2-pyrazine-, 6-methoxypyrazine-, and 2-quinolinecarbimidate. An important element of the chosen synthetic route is that there is no need for isolation of carbimidates and they are used in situ after generation from the corresponding carbonitriles in methanol in the presence of DBU (1,8-diazabicycloundec-7-ene). Isolated carbimidates are easy to obtain in pure form and can be also used for 2-pyrazine, 6-chloropyrazine, and 6-methoxypyrazine derivatives. The carbimidates mentioned above underwent reaction with 2-chlorobenzenesulfonamide and 4-chloropyridine-3-sulfonamide. The reactions were carried out in a methanol solution of DBU. That led to the formation of amidine structures with the 2-chlorobenzenesulfonyl or 4-chloropyridine-3-sulfonyl substituent (**1**–**12**, **15**, **16**; Scheme 1). The amidine structures **9**, **10**, **13**, and **14** were prepared from pure isolated carbimidates by refluxing equimolar amounts of the reagents in diglyme (bis(2-methoxyethyl)ether).

**Figure:**
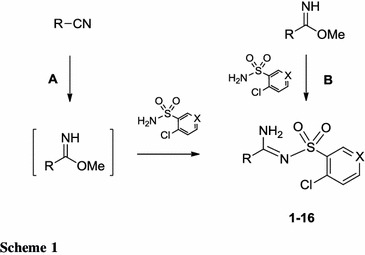

**Fig. 1:**
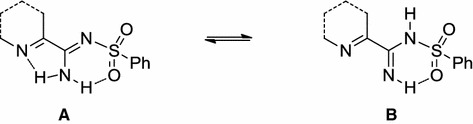
Possible tautomeric structures of sulfonyl-carboximidamides

In the ^1^H NMR spectra of the target amidines the signals for all the protons of aromatic systems were observed and two signals for the NH groups were shifted from each other at about 1 ppm. These separated signals could be due to the amino-imine structure adopted by the obtained derivatives (Fig. 1, structure B), as suggested by Northey and co-workers [20]. They could also be the result of the magnetic inequivalence of NH protons in the amine moiety upon formation of a hydrogen bond in the case of heterocyclic compounds in which the amidine group is in the α position to the nitrogen atom of the heterocyclic ring (structure A), as shown in the previous article [21].

**Fig. 2:**
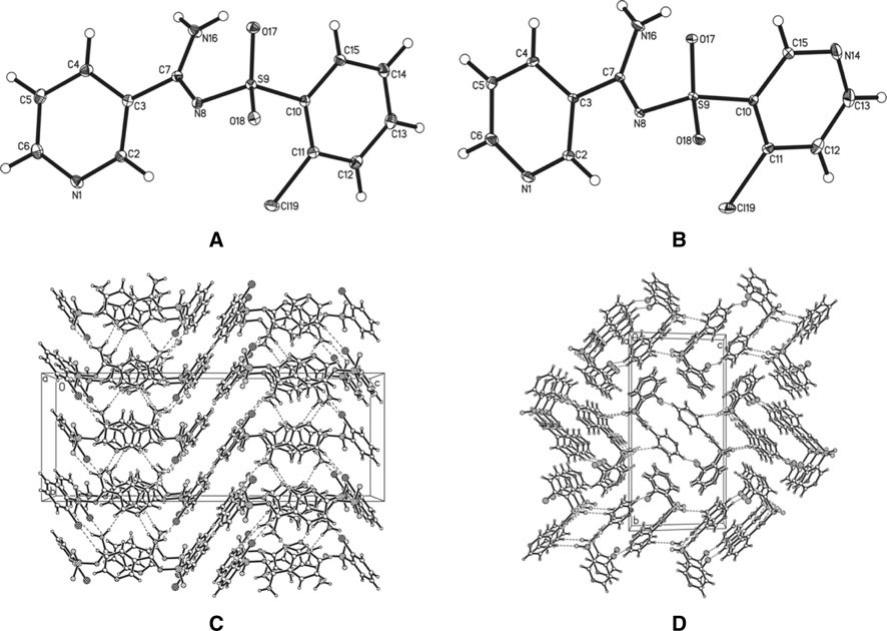
Structure of **3** (**a**) and **4** (**b**) showing 25 % probability displacements for ellipsoid. H atoms shown as small spheres of arbitrary radius. The arrangement of the molecules in the crystal structure of **3** (**c**) and **4** (**d**) viewed along *b* axis. *Dashed lines* N–H···O, N–H···N, C–H···Cl, and C–H···O interactions

Such a structure could also be stabilized by hydrogen bonding between the second proton of the amine group and the oxygen atom of the sulfonyl moiety. This issue was resolved by X-ray studies performed on derivatives **3** and **4**. The obtained results revealed the tautomeric structure A (Fig. 2a, b). Thus, formation of hydrogen bonds is the reason for the magnetic inequivalence of the protons of the amino group and separate signals in the ^1^H NMR spectra of the synthesized compounds.

### Crystal structure of compounds **3** and **4**

In the molecules of the title compounds (Fig. 2a, b) the bond lengths and angles characterizing the geometry of the pyridine skeleton, amino and sulfonyl groups, and benzene ring are typical for this group of compounds [21], although earlier reports from other authors suggested the existence of imine tautomeric structures. Comparison of molecules of **3** and **4** shows that the structures of both compounds are very similar (Table 1).

**Table 1:** Crystal data and structure refinement for compounds **3** and **4**

	Compound **3**	Compound **4**
Empirical formula	C_12_H_10_ClN_3_O_2_S	C_11_H_9_ClN_4_O_2_S
Formula weight	295.74	296.73
Temperature/K	295(2)	295(2)
Wavelength/Å	1.54184	1.54184
Crystal system	Orthorhombic	Monoclinic
Space group	*P* *bca*	*P*2_1_/*n*
Unit cell dimensions		
*a*/Å	7.7435(2)	5.5337(5)
*b*/Å	10.9172(2)	20.8163(9)
*c*/Å	29.3315(6)	10.6688(6)
*β*/°		104.298(8)
*V*/Å^3^	2,479.61(9)	1,190.88(14)
*Z*	8	4
*D*_calcd_/mg m^−3^	1.584	1.655
Absorption coefficient/mm^−1^	4.330	4.536
*F*(000)	1,216	608
Crystal size/mm^3^	0.40 × 0.12 × 0.10	0.34 × 0.18 × 0.13
*Θ* range for data collection/deg	3.01–67.18	4.25–67.35
Limiting indices	−7 ≤ *h* ≤ 8, −13 ≤ *k* ≤ 13, −34 ≤ *l* ≤ 34	−5 ≤ *h* ≤ 6, −24 ≤ *k* ≤ 24, −12 ≤ *l* ≤ 12
Reflections collected/unique	7,923/2,179 [*R*_int_ = 0.0581]	5,600/20,929 [*R*_int_ = 0.0961]
Completeness 2*Θ* = 50.00°/%	98.2	97.6
Refinement method	Full-matrix least-squares on *F* ^2^	
Data/restraints/parameters	2,179/0/172	2,092/0/178
Goodness-of-fit on *F* ^2^	1.079	1.058
Final *R* indices [*I* > 2*σ*(*I*)]	*R*_1_ = 0.0523 *wR*_2_ = 0.1316	*R*_1_ = 0.0645 *wR*_2_ = 0.1567
*R* indices (all data)	*R*_1_ = 0.0555 *wR*_2_ = 0.1352	*R*_1_ = 0.1037 *wR*_2_ = 0.1811
Largest diff. peak and hole/e Å^−3^	0.313 and −0.494	0.352 and −0.580

However, we can observe differences in the dihedral angles and molecular interactions in the crystal packing. With respective average deviations from planarity of 0.006(1) and 0.007(3) Å, the nearly planar pyridine ring and aromatic fragment (2-chlorobenzene in compound **3** and 2-chloro-3-pyridine in compound **4**) are oriented at an angle of 60.6(2) and 69.1(3)° to each other, for I and II, respectively. In the packing of both compounds, the amino group participates in the intramolecular N–H···O and intermolecular N–H···O interactions (Fig. 2c, d). This group is also engaged in N–H···N intermolecular interactions, where the acceptor of the H atom is the endocyclic N atom from the pyridine (compound **3**) or chloropyridine (compound **4**) rings. The neighboring molecules in **3** and **4** are also linked through the C–H···Cl hydrogen bond. Additionally, weak C–H···O hydrogen bonds are observed in the crystal packing of **4**.

### Biological activity

Four of the obtained sulfonyl-carboximidamides (**2**, **7**, **8**, **10**) were evaluated for their in vitro tuberculostatic activity against the *Mycobacterium tuberculosis* H_37_Rv strain and two “wild” strains isolated from tuberculosis patients: one (sp. 210) resistant to *p*-aminosalicylic acid (PAS), isonicotinic acid hydrazide (INH), ethambutol (ETB), and rifampicin (RFP), and the another (sp. 192) fully sensitive to the administrated tuberculostatics. Isoniazid (INH) was used as a reference drug. The tested compounds showed rather weak tuberculostatic activity, weaker than the reference INH (MIC 0.5–1.0 μg/cm^3^). For all the compounds the determined MIC values were 25–50 μg/cm^3^ against the three tested strains (Table 2).

**Table 2:** Antibacterial activity of the synthesized compounds against *P. acnes* and *M. tuberculosis*

No.	MIC/μg/cm^3^			
	*P. acnes* ^a^	*M. tuberculosis*		
		H_37_Rv	sp. 192	sp. 210
**1**	25	–	–	–
**2**	50	50	50	50
**3**	>100	–	–	–
**4**	>100	–	–	–
**5**	>100	–	–	–
**6**	>100	–	–	–
**7**	>100	50	50	50
**8**	>100	50	50	50
**9**	>100	–	–	–
**10**	>100	50	25	50
**11**	>100	–	–	–
**12**	>100	–	–	–
**13**	>100	–	–	–
**14**	>100	–	–	–
**15**	12.5	–	–	–
**16**	>100	–	–	–
INH	–	0.5	0.5	1.1

Minimum inhibitory concentrations for *P. acnes* were assessed optically as the lowest concentration of a test material which caused no bacteria growth. MICs for *M. tuberculosis* strains were determined by twofold serial dilution method for microdilution plates and for mycobacterial strains by twofold classical test-tube method of successive dilution

*INH* isoniazid,* –* not tested

^a^ *P. acnes* ATCC 11827

The compounds were also tested for their antibacterial activity against *P. acnes* (ATCC 11827) and *Brevibacterium linens* (ATCC 9174). All of the synthesized sulfonyl-carboximidamides (**1**–**13**) exhibited activity with MICs greater than 256 μg/cm^3^, which meant that those values did not fit standard test concentrations. All synthesized compounds were tested on *B. linens* but no test compound had a MIC of less than 100 μg/cm^3^. The compounds were then tested on *P. acnes*. Only three of the tested compounds (**1**, **2**, **15**) exhibited moderate antibacterial activity with MIC values of 12.5–50 μg/cm^3^. For all other compounds the MIC values were above 100 μg/cm^3^. Compound **15** was the most active (MIC 12.5 μg/cm^3^).

**Fig. 3:**
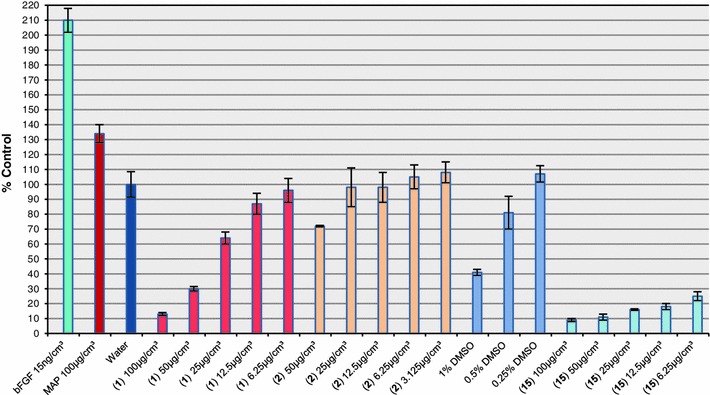
Total cell/protein count for compounds **1**, **2**, and **15**

The most antibacterially potent sulfonylcarboximidamides **1**, **2**, and **15** were then tested for their effects on the proliferation of neonatal human dermal fibroblasts (ATCC PCS-201-010). MAP (magnesium ascorbyl phosphate) and bFGF (basic fibroblast growth factor) were used as the positive control (Fig. 3). Compound **2** had no cytotoxic activity. Irrespective to the compound concentration the cell growth remained at the level corresponding to the water-treated control. Compound **1** had a weak inhibitory activity. For low compound concentrations (6.25–12.5 μg/cm^3^) no cytotoxic effect was evident. In the range 25–100 μg/cm^3^ a linear relation of the cytotoxic effect was observed with an 88 % growth inhibitory activity at a concentration of 100 μg/cm^3^. Compound **15** was strongly cytotoxic, even at the lowest concentration tested (6.25 μg/cm^3^—70 % growth inhibitory activity).

In view of the cytotoxic activity of compound **15**, it was of interest to determine whether those compounds had an antitumor potential. Compounds **2**–**16** were tested in the framework of the Development Therapeutic Program (DTP) at the National Cancer Institute (Bethesda, MD, USA) on a panel of 60 human tumor cell lines derived from nine different cancer types: leukemia, lung, colon, CNS, melanoma, ovarian, renal, prostate, and breast. Among the compounds **2**–**16** tested in the preliminary NCI-60 one-dose screen test seven of them (44 %) exhibited distinct growth inhibition (ΔGI) properties (Table 3). Three compounds (**2**, **7**, **11**) were active towards one renal cancer UO-31 cell line. These compounds inhibited the growth of that cell line with ΔGI of from 19.4 to 21.1 %. Derivatives **4**, **10**, and **12** exhibited activity against two cell lines. Compound **4** was potent towards the melanoma MALME-3M cell line (ΔGI 20.0 %) and the renal cancer A498 cell line (ΔGI 24.1 %). Compound **10** exhibited activity against CNS cancer SNB-75 cell line (ΔGI 19.3 %) and compound **11** against melanoma MALME-3M cell line (ΔGI 22.6 %). Derivative **12** was active towards two cell lines, non-small cell lung cancer HOP-92 (ΔGI 25.9 %) and NCI-H522, against which the compound exhibited cytotoxic activity (ΔGI 142.9 %). Derivative **15** was the most potent compound. That compound exhibited activity towards eleven cell lines: leukemia HL-60 (ΔGI 19.7 %) and K-562 (ΔGI 35.9 %), non-small cell lung cancer NCI-H460 (ΔGI 19.6 %), colon cancer HT29 (ΔGI 60.4 %), KM12 (ΔGI 23.0 %) and SW-620 (ΔGI 24.7 %), melanoma MDA-MB-435 (ΔGI 80.4 %), ovarian cancer NCI/ADR RES (ΔGI 42.9 %), renal cancer TK-10 (ΔGI 21.7 %), breast cancer MCF7 (ΔGI 15.7 %) and MDA-MB-468 (ΔGI 42.2 %).

**Table 3:** One-dose screening data of in vitro tumor growth inhibition for compounds **2**, **4**, **7**, **10**–**12**, and **15** at a dose of 10 μM

No.	Growth percent mean values (MG_MID^a^)/%	Panel	Cell line	Growth inhibition ΔGI/%
**2**	98.2	Renal cancer	UO-31	20.4
**4**	104.8	Melanoma	MALME-3 M	20.0
		Renal cancer	A498	24.1
**7**	103.0	Renal cancer	UO-31	19.4
**10**	101.9	CNS cancer	SNB-75	19.3
**11**	102.1	Renal cancer	UO-31	22.6
**12**	97.8	Non-small cell lung cancer	HOP-92	25.9
			NCI-H522	142.9^b^
**15**	53.4	Leukemia	HL-60	19.7
			K-562	35.9
		Non-small cell lung cancer	NCI-H460	19.6
		Colon cancer	HT29	60.4^b^
			KM12	23.0
			SW-620	24.7
		Melanoma	MDA-MB-435	80.4^b^
		Ovarian cancer	NCI/ADR-RES	42.9
		Renal cancer	TK-10	21.7
		Breast cancer	MCF7	15.7
			MDA-MB-468	42.2

Data obtained from the NCI-60 DTP human tumor cell line screening

^a^MIG_MID mean graph midpoint, i.e., arithmetical mean value of growth percent for all tested cell lines

^b^Cytotoxic effect (lethality)

Compound **15** was selected for further studies in five concentrations in the range of 10^−4^ to 10^−8^ M. The activity of the compound was expressed by three dose–response parameters: *GI*_50_—the molar concentration that inhibits 50 % net cell growth, TGI—the molar concentration leading to total growth inhibition, *LC*_50_—molar concentration leading to 50 % net cell death (Table 4). Derivative **15** was the most potent towards the leukemia SR cell line (*GI*_50_ 0.92 μM), the non-small cell lung cancer NCI-H522 (*GI*_50_ 2.28 μM), the CNS cancer SNB-75 cell line (*GI*_50_ 2.52 μM), the melanoma MDA-MB-435 cell line (*GI*_50_ 1.13 μM), the ovarian cancer NCI/ADR-RES cell line (*GI*_50_ 2.32 μM), and the breast cancer MDA-MB-468 cell line (*GI*_50_ 2.01 μM). In general, compound **15** exhibited the highest activity against leukemia cell lines. The *GI*_50_ mean value for that cell line panel was 3.52 μM.

**Table 4:** Inhibition of in vitro human cancer cell lines growth by compound** 15**

Panel/cell line	*GI*_50_^a^/μM	TGI^b^/μM
Leukemia		
CCRF-CEM	3.65	^c^
HL-60 (TB)	2.29	8.41
K-562	3.11	^c^
MOLT-4	5.91	^c^
RPMI-8226	5.27	^c^
SR	0.92	21.40
Non-small cell lung cancer		
A549/ATCC	5.37	^c^
EKVX	8.75	^c^
HOP-62	5.37	^c^
HOP-92	9.34	90.80
NCI-H226	10.80	^c^
NCI-H23	5.44	^c^
NCI-H322M	5.19	^c^
NCI-H460	4.02	^c^
NCI-H522	2.28	7.62
Colon cancer		
COLO 205	3.68	16.00
HCC-2998	9.63	59.40
HCT-116	4.66	^c^
HCT-15	4.23	^c^
HT29	3.40	14.90
KM12	3.29	^c^
SW-620	3.45	^c^
CNS cancer		
SF-268	6.79	^c^
SF-295	3.83	86.30
SF-539	4.19	^c^
SNB-19	5.61	^c^
SNB-75	2.52	30.60
U251	4.48	^c^
Melanoma		
LOX IMVI	4.05	^c^
MALME-3M	5.42	^c^
M-14	3.61	^c^
MDA-MB-435	1.13	3.78
SK-MEL-2	3.40	^c^
SK-MEL-28	5.45	12.00
SK-MEL-5	3.31	50.50
UACC-257	11.70	^c^
UACC-62	6.07	^c^
Ovarian cancer		
IGROV1	9.42	^c^
OVCAR-3	9.27	3.78
OVCAR-4	9.15	9.53
OVCAR-5	7.71	^c^
OVCAR-8	5.74	12.00
NCI/ADR-RES	2.32	50.50
SK-OV-3	3.93	^c^
Renal cancer		
786-0	4.94	^c^
A498	8.08	^c^
ACHN	7.19	13.10
CAKI-1	5.21	^c^
RXF 393	4.02	35.90
SN 12C	7.67	^c^
TK-10	6.02	7.08
UO-31	7.26	48.50
Prostate cancer		
PC-3	9.59	^c^
DU-145	4.96	^c^
Breast cancer		
MCF-7	3.28	^c^
MDA-MB-231/ATCC	13.00	^c^
HS 578T	8.98	^c^
BT-549	5.08	56.00
T-47D	7.04	^c^
MDA-MB-468	2.01	6.19

Data obtained from the NCI-60 DTP human tumor cell line screening

Omitted *LC*_50_ values (molar concentration leading to 50 % net cell death) were >100 μM except SK-MEL-5 cell line in melanoma panel (36.2 μM)

^a^ *GI*_50_ the molar concentration that inhibits 50 % net cell growth

^b^TGI the molar concentration leading to total growth inhibition

^c^Determined TGI values were >100 μM

## Conclusion

A series of novel heterocyclic sulfonyl-carboximidamides with different nitrogen heterocyclic systems were synthesized successfully via condensation of heterocyclic methyl carbimidates with 2-chlorobenzenesulfonamide and 4-chloropyridine-3-sulfonamide. Structures of all these new compounds were confirmed by IR and NMR spectra as well as elemental analyses. X-ray crystallography of compounds **3** and **4** demonstrated the presence of the amine tautomeric structure. The antimicrobial activities of the synthesized compounds were evaluated against *P. acnes* and *B. linens* as well as *M. tuberculosis*. The results showed that the synthesized sulfonamide derivatives exhibited rather poor antimicrobial activities in vitro. Seven compounds (**2**, **4**, **7**, **10**–**12**, **15**) were able to inhibit the growth of some cancer cell lines, whereas the 2-quinoline derivative **15** showed the highest activity with *GI*_50_ values of from 0.92 to 13.00 μM.

## Experimental

All materials and solvents were of analytical reagent grade. Thin-layer chromatography was performed on Merck silica gel 60F_254_ plates and visualized with UV. The results of elemental analyses (% C, H, N) for all obtained compounds were in agreement with calculated values within ±0.3 %. ^1^H NMR spectra in DMSO-*d*_6_ were recorded on Varian Unity Plus (500 MHz) and Varian Gemini (200 MHz) instruments (Varian, Palo Alto, CA). IR spectra were determined as KBr pellets of the solids on a Satellite FT-IR spectrophotometer (Mattson Instruments, Madison, WI). Electrospray MS analyses for compounds **1**, **6**, **9**, **14**, and **16** were performed on an HCT Ultra Bruker Daltonics spectrometer operating in positive- and negative-ion modes (sheath gas N_2_, temperature 300 °C, flow 7 dm^3^/min, pressure 10 psi (689.48 hPa); capillary voltage in positive ion mode +4 kV, in negative ion mode −4 kV). Compound samples were prepared in acetonitrile (**1**, **9**, **14**, **16**) or methanol (**6**). Melting points were determined with a Boethius apparatus (Franz Küstner Nachf. KG, Dresden, Germany). Methyl pyrazine-2-carbimidate and methyl 6-methoxypyrazine-2-carbimidate required for syntheses of compounds **9**, **10** and **13**, **14** were obtained according to the method described earlier by Foks and co-workers [22, 23]. Reaction yield and compound characteristics were found to be identical with those described (m.p. 115–116 and 100–101 °C, respectively).

### General method A for the synthesis of sulfonyl-carboximidamides **1**–**12**, **15**, **16**

The respective carbonitrile (5 mmol) was dissolved in 10 cm^3^ of methanol and 0.1 cm^3^ (0.7 mmol) of DBU was added. The mixture was refluxed for 0.5 h required for methyl carbimidate formation. Then 2-chlorobenzenesulfonamide or 4-chloropyridine-3-sulfonamide (4 mmol) was added. The mixture was refluxed for another 3 h. The solvent was evaporated under vacuum and 30 g of ice was added. The precipitate was filtered and recrystallized from a suitable solvent.

### Alternative method B for the synthesis of sulfonyl-carboximidamides **9**, **10**, **13**, **14**

Methyl pyrazine-2-carbimidate or methyl 6-methoxypyrazine-2-carbimidate (3 mmol) and the respective sulfonamide (3 mmol) were refluxed in 5 cm^3^ of diglyme for 15 min. Then the mixture was cooled and 30 g of ice was added. The precipitate was filtered and recrystallized from a suitable solvent with addition of activated carbon.

#### *N′*-*[(2*-*Chlorophenyl)sulfonyl]*-*2*-*pyridinecarboximidamide* (**1**, C_12_H_10_ClN_3_O_2_S)

The crude product was recrystallized from methanol affording 1.1 g (93 %) **1**. M.p.: 140–142 °C; IR (KBr): $\bar{\nu }$ = 3,423, 3,211 (ν N–H), 1,613 (ν C=N), 1,583 (ν C=C), 1,537 (ν N–H), 1,292, 1,284, 1,157 (ν SO_2_), 1,041 (δ C–H), 828, 759 (γ C–H), 587 (γ N–H) cm^−1^; ^1^H NMR (200 MHz): δ = 7.51–7.70 (m, 4H, 2H Ph and 2H pyridine), 7.95–8.18 (m, 3H, 2H Ph and 1H pyridine), 8.40 (br s, 1H, NH + D_2_O exchangeable), 8.70 (d, *J* = 4.7 Hz, 1H, pyridine), 9.18 (br s, 1H, NH + D_2_O exchangeable) ppm; ^13^C NMR (50 MHz): δ = 123.26, 127.75, 127.92, 129.60, 131.56, 131.60, 134.13, 138.46, 139.63, 148.69, 149.28, 159.76 ppm; MS (−): *m*/*z* = 611 (100 %, [2M+Na−3H]^−^), 294 (32 %, [M−2H]^−^), 258 (28 %, [M−2H–Cl]^−^); MS (+): *m*/*z* = 613 (25 %, [2M+Na−H]^+^), 318 (100 %, [M+Na−H]^+^), 298 (71 %, [M+2H]^+^).

#### *N′*-*[(4*-*Chloropyridin*-*3*-*yl)sulfonyl]*-*2*-*pyridinecarboximidamide* (**2**, C_11_H_9_ClN_4_O_2_S)

This compound was recrystallized from ethanol affording 0.82 g (69 %) **2**. M.p.: 150–152 °C; IR (KBr): $\bar{\nu }$ = 3,430, 3,390, 3,323 (ν N–H), 1,629 (ν C=N), 1,587 (ν C=C), 1,559 (ν N–H), 1,297, 1,275, 1,151 (ν SO_2_), 1,115, 833 (δ C–H), 592 (γ N–H) cm^−1^; ^1^H NMR (200 MHz): δ = 7.67 (t, *J* = 5.9 Hz, 1H, pyridine), 7.96–8.13 (m, 2H, 1H pyridine and 1H 4-chloropyridine), 8.57 (br s, 1H, NH + D_2_O exchangeable), 8.70–8.77 (m, 2H, 1H pyridine and 1H 4-chloropyridine), 9.22 (s, 1H, 4-chloropyridine), 9.32 (br s, 1H, NH + D_2_O exchangeable) ppm; ^13^C NMR (50 MHz): δ = 123.41, 126.75, 128.04, 135.75, 138.50, 141.92, 148.50 (2C), 149.38, 154.45, 160.03 ppm.

#### *(Z)-N′-[(2-Chlorophenyl)sulfonyl]-3-pyridinecarboximidamide* (**3**, C_12_H_10_ClN_4_O_2_S_2_)

This compound was recrystallized from dioxane affording 0.51 g (43 %) **3**. M.p.: 188–190 °C; IR (KBr): $\bar{\nu }$ = 3,341 (ν N–H), 3,156 (ν C–H), 1,644 (ν C=N), 1,591 (ν C=C), 1,546 (δ N–H), 1,295, 1,281, 1,162, 1,146 (ν SO_2_), 1,042 (δ C–H), 839, 761 (γ C–H), 591 (γ N–H) cm^−1^; ^1^H NMR (200 MHz): δ = 7.49–7.65 (m, 4H, 2H Ph and 2H pyridine), 8.12–8.21 (m, 2H, Ph), 8.50 (br s, 1H, NH + D_2_O exchangeable), 8.74 (d, *J* = 4.4 Hz, 2H, pyridine), 9.00 (s, 1H, pyridine), 9.39 (br s, 1H, NH + D_2_O exchangeable) ppm; ^13^C NMR (50 MHz): δ = 123.76, 127.65 (2C), 129.59, 129.71, 131.49, 131.97, 133.99, 136.04, 139.81, 149.03, 153.09, 161.94 ppm.

#### *(Z)-N′-[(4-Chloropyridin-3-yl)sulfonyl]-3-pyridinecarboximidamide* (**4**, C_11_H_9_ClN_4_O_2_S)

This compound was recrystallized from dioxane affording 0.36 g (30 %) **4**. M.p.: 192–194 °C; IR (KBr): $\bar{\nu }$ = 3,439, 3,330 (ν N–H), 1,620 (ν C=N), 1,554, 1,514 (ν C=C), 1,283, 1,146 (ν SO_2_), 836, 795 (γ C–H), 592 (γ N–H) cm^−1^; ^1^H NMR (200 MHz): δ = 7.49–7.56 (m, 1H, pyridine), 7.78 (d, *J* = 5.4 Hz, 1H, 4-chloropyridine), 8.17–8.19 (m, 1H, pyridine), 8.22 (br s, 1H, NH + D_2_O exchangeable), 8.74–8.77 (m, 2H, 1H pyridine and 1H 4-chloropyridine), 9.00 (d, *J* = 2.4 Hz, 1H, pyridine), 9.20 (s, 1H, 4-chloropyridine), 9.48 (br s, 1H, NH + D_2_O exchangeable) ppm; ^13^C NMR (50 MHz): δ = 124.08, 126.78, 129.60, 135.75 (2C), 141.93, 148.58, 149.38, 152.60, 154.39, 162.03 ppm.

#### *N′-[(2-Chlorophenyl)sulfonyl]-4-pyridinecarboximidamide* (**5**, C_12_H_10_ClN_3_O_2_S)

This compound was recrystallized from methanol/water (1:1) affording 0.65 g (55 %) **5**. M.p.: 182–185 °C; IR (KBr): $\bar{\nu }$ = 3,387 (ν N–H), 3,175 (ν C–H), 1,658 (ν C=N), 1,581 (ν C=C), 1,526 (δ N–H), 1,289, 1,145 (ν SO_2_), 850, 749 (γ C–H), 591 (γ N–H) cm^−1^; ^1^H NMR (500 MHz): δ = 7.56 (t, *J* = 7.8 Hz, 1H, Ph), 7.65 (m, 2H, Ph), 7.75 (d, *J* = 5.4 Hz, 1H, pyridine), 8.14 (d, *J* = 7.3 Hz, 1H, Ph), 8.56 (br s, 1H, NH + D_2_O exchangeable), 8.74 (d, *J* = 5.9 Hz, 1H, pyridine), 9.42 (br s, 1H, NH + D_2_O exchangeable) ppm; ^13^C NMR (50 MHz): δ = 121.92 (2C), 127.70, 129.56, 131.53, 131.99, 134.11, 139.61, 141.12, 150.59 (2C), 161.79 ppm.

#### *N′-[(4-Chloropyridin-3-yl)sulfonyl]-4-pyridinecarboximidamide* (**6**, C_11_H_9_ClN_4_O_2_S)

This compound was recrystallized from dioxane affording 0.71 g (60 %) **6**. M.p.: 215–218 °C; IR (KBr): $\bar{\nu }$ = 3,385, 3,330 (ν N–H), 3,088, 2,964 (ν C–H), 1,660 (ν C=N), 1,570 (ν C=C), 1,533 (δ N–H), 1,280, 1,116 (ν SO_2_), 854, 769 (γ C–H), 594 (γ N–H) cm^−1^; ^1^H NMR (200 MHz): δ = 7.76–7.80 (m, 3H, 2H pyridine and 1H 4-chloropyridine), 8.72–8.77 (m, 4H, 2H pyridine and 1H 4-chloropyridine and 1H NH + D_2_O exchangeable), 9.19 (s, 1H, 4-chloropyridine), 9.55 (br s, 1H, NH + D_2_O exchangeable) ppm; ^13^C NMR (50 MHz): δ = 122.52 (2C), 126.53, 135.64, 141.84, 141.95, 149.34 (2C), 149. 67, 154.48, 161.86 ppm; MS (−): *m*/*z* = 297 (26 %, [M]^−^), 295 (66 %, [M−2H]^−^), 259 (100 %, [M−2H–Cl]^−^); MS (+): *m*/*z* = 297 (70 %, [M]^+^), 153 (100 %, [M−H–Cl–C_5_H_3_N–NH_2_–O]^+^).

#### *N′-[(2-Chlorophenyl)sulfonyl]pyrimidine-2-carboximidamide* (**7**, C_11_H_9_ClN_4_O_2_S)

This compound was recrystallized from methanol affording 0.99 g (83 %) **7**. M.p.: 191–192 °C; IR (KBr): $\bar{\nu }$ = 3,396, 3,309 (ν N–H), 1,623 (ν C=N), 1,554, 1,391 (ν C=C), 1,279, 1,151 (ν SO_2_), 838, 693 (γ C–H), 589 (γ N–H) cm^−1^; ^1^H NMR (200 MHz): δ = 7.51–7.65 (m, 3H, Ph), 7.70 (t, *J* = 5.0 Hz, 1H, pyrimidine), 8.13 (d, *J* = 7.2 Hz, 2H, Ph), 8.54 (br s, 1H, NH + D_2_O exchangable), 9.00 (d, *J* = 5.0 Hz, 2H, pyrimidine), 9.15 (br s, 1H, NH + D_2_O exchangable) ppm; ^13^C NMR (50 MHz): δ = 123.73, 127.73, 129.49, 131.66, 131.89, 134.08, 139.76, 158.20 (2C), 158.89, 159.83 ppm.

#### *N′-[(4-Chloropyridin-3-yl)sulfonyl]pyrimidine-2-carboximidamide* (**8**, C_10_H_8_ClN_5_O_2_S)

This compound was recrystallized from dioxane affording 0.62 g (52 %) **8**. M.p.: 162–163 °C; IR (KBr): $\bar{\nu }$ = 3,288 (ν N–H), 1,623 (ν C=N), 1,560, 1,397 (ν C=C), 1,291, 1,219, 1,147 (ν SO_2_), 840 (γ C–H), 600 (γ N–H) cm^−1^; ^1^H NMR (200 MHz): δ = 7.69–7.77 (m, 2H, 1H pyrimidine and 1H 4-chloropyridine), 8.75 (d, *J* = 4.3 Hz, 1H, 4-chloropyridine), 8.85 (br s, 1H, NH + D_2_O exchangable), 8.97 (d, *J* = 4.5 Hz, 2H, pyrimidine), 9.14 (s, 1H, 4-chloropyridine), 9.39 (br s, 1H, NH + D_2_O exchangeable) ppm; ^13^C NMR (50 MHz): δ = 123.73, 126.67, 136.07, 141.94, 149.25, 154.26, 158.37 (2C), 160.53, 161.25 ppm.

#### *N′-[(2-Chlorophenyl)sulfonyl]pyrazine-2-carboximidamide* (**9**, C_11_H_9_ClN_4_O_2_S)

This compound was recrystallized from ethanol affording 0.55 g (46 %) **9** for method A and 0.68 g (76 %) for method B. M.p.: 173–174 °C; IR (KBr): $\bar{\nu }$ = 3,438, 3,356, 3,329, 3,254 (ν N–H), 3,092 (ν C–H), 1,615 (ν C=N), 1,337, 1,278, 1,182, 1,153 (ν SO_2_), 1,041 (δ C–H), 592 (γ N–H) cm^−1^; ^1^H NMR (500 MHz): δ = 7.57–7.59 (m, 1H, Ph), 7.66–7.67 (m, 2H, Ph), 8.18 (d, *J* = 7.3 Hz, 1H, Ph), 8.53 (br s, 1H, NH + D_2_O exchangeable), 8.20 (t, *J* = 2.4 Hz, 1H, pyrazine), 8.81 (d, *J* = 2.4 Hz, 1H, pyrazine), 9.20 (d, *J* = 1.4 Hz, 1H, pyrazine), 9.34 (br s, 1H, NH + D_2_O exchangable) ppm; ^13^C NMR (50 MHz): δ = 127.76, 129.69, 131.58, 132.03, 134.22, 139.42, 144.03, 144.28 (2C), 148.58, 158.79 ppm; MS (−): *m*/*z* = 613 (92 %, [2M+Na−3H]^−^), 297 (43 %, [M]^−^), 295 (100 %, [M−2H]^−^), 259 (79 %, [M−2H–Cl]^−^); MS (+): *m*/*z* = 615 (29 %, [2M+Na−H]^+^), 319 (100 %, [M+Na−H]^+^), 153 (71 %, [M−Cl–C_6_H_4_–NH_2_–O]^+^).

#### *N′*-*[(4*-*Chloropyridin*-*3*-*yl)sulfonyl]pyrazine*-*2*-*carboximidamide* (**10**, C_10_H_8_ClN_5_O_2_S)

This compound was recrystallized from ethanol affording 0.79 g (66 %) **10** for method A and 0.38 g (42 %) for method B. M.p.: 187–188 °C; IR (KBr): $\bar{\nu }$ = 3,380, 3,239 (ν N–H), 1,637 (ν C=N), 1,560 (ν C=C), 1,547 (δ N–H), 1,285, 1,153, 1,120 (ν SO_2_), 853, 793 (γ C–H), 600 (γ N–H) cm^−1^; ^1^H NMR (200 MHz): δ = 7.80 (d, *J* = 4.8 Hz, 1H, 4-chloropyridine), 8.57 (br s, 1H, NH + D_2_O exchangeable), 8.77–8.80 (m, 2H, 1H pyrazine and 1H 4-chloropyridine), 8.92 (d, *J* = 3.2 Hz, 1H, 4-chloropyridine), 9.21–9.23 (m, 2H, pyrazine), 9.43 (br s, 1H, NH + D_2_O exchangable) ppm; ^13^C NMR (50 MHz): δ = 126.79, 135.57, 141.98, 144.03, 144.43 (2C), 148.68, 149.44, 154.53, 159.11 ppm.

#### *6*-*Chloro*-*N′*-*(2*-*chlorophenylsulfonyl)pyrazine*-*2*-*carboximidamide* (**11**, C_11_H_8_Cl_2_N_5_O_2_S)

This compound was recrystallized from dioxane/methanol (1:1) affording 1.1 g (85 %) **11**. M.p.: 195–198 °C; IR (KBr): $\bar{\nu }$ = 3,394, 3,288, 3,236 (ν N–H), 1,642 (ν C=N), 1,554 (ν C=C), 1,522 (δ N–H), 1,362, 1,297, 1,152, 11,07 (ν SO_2_), 892, 790, 757 (γ C–H), 567 (γ N–H) cm^−1^; ^1^H NMR (500 MHz): δ = 7.56–7.59 (m, 1H, Ph), 7.64–7.69 (m, 2H, Ph), 8.18 (d, *J* = 7.8 Hz, 1H, Ph), 8.56 (br s, 1H, NH + D_2_O exchangeable), 9.07 (s, 1H, pyrazine), 9.13 (s, 1H, pyrazine), 9.34 (br s, 1H, NH + D_2_O exchangeable) ppm; ^13^C NMR (50 MHz): δ = 127.77, 129.69, 132.04, 134.30, 139.27, 142.45, 144.56, 147.52, 148.42, 157.80, 158.70 ppm.

#### *6-Chloro-N′-[(4-chloropyridin-3-yl)sulfonyl]pyrazine-2-carboximidamide* (**12**, C_10_H_7_Cl_2_N_5_O_2_S)

This compound was recrystallized from methanol/water (1:1) affording 0.64 g (48 %) **12**. M.p.: 160–162 °C; IR (KBr): $\bar{\nu }$ = 3,372 (ν N–H), 3,092 (ν C–H), 1,656 (ν N=C), 1,563, 1,547 (ν C=C), 1,518 (δ N–H), 1,368, 1,302, 1,150 (ν SO_2_), 796 (γ C–H), 601 (γ N–H) cm^−1^; ^1^H NMR (500 MHz): δ = 7.80 (d, *J* = 5.4 Hz, 1H, 4-chloropyridine), 8.76 (br s, 1H, NH + D_2_O exchangeable), 8.78 (d, *J* = 5.4 Hz, 1H, 4-chloropyridine), 9.07 (s, 1H, pyrazine), 9.16 (s, 1H, 4-chloropyridine), 9.22 (s, 1H, pyrazine), 9.48 (br s, 1H, NH + D_2_O exchangeable) ppm; ^13^C NMR (50 MHz): δ = 126.80, 135.48, 142.03, 142.61, 144.45, 147.49, 148.49, 149.42, 154.55, 158.13 ppm.

#### *N′*-*(2*-*Chlorophenylsulfonyl)*-*6*-*methoxypyrazine*-*2*-*carboximidamide* (**13**, C_12_H_11_ClN_4_O_3_S)

This compound was recrystallized from methanol affording 0.75 g (76 %) **13**. M.p.: 227–230 °C; IR (KBr): $\bar{\nu }$ = 3,401, 3,248 (ν N–H), 1,643 (ν C=N), 1,541 (ν C=C), 1,381, 1,320, 1,281, 1,146, 1,106 (ν SO_2_), 804 (γ C–H), 584, 561 (γ N–H) cm^−1^; ^1^H NMR (200 MHz): δ = 4.03 (s, 3H, OCH_3_), 7.51–7.65 (m, 3H, Ph), 8.15–8.19 (m, 1H, Ph), 8.55 (s, 1H, pyrazine), 8.73 (s, 1H, pyrazine), 8.80 (br s, 1H, NH + D_2_O exchangeable), 9.00 (br s, 1H, NH + D_2_O exchangeable) ppm; ^13^C NMR (50 MHz): δ = 54.59, 127.73, 129.61, 131.55, 132.03, 134.16, 135.74, 139.81 (2C), 140.87, 158.70, 159.08 ppm.

#### *N′*-*[(4*-*Chloropyridin*-*3*-*yl)sulfonyl]*-*6*-*methoxypyrazine*-*2*-*carboximidamide* (**14**, C_11_H_10_ClN_5_O_3_S)

This compound was recrystallized from DMSO affording 0.32 g (33 %) **14**. M.p.: 265–270 °C (decomp.); IR (KBr): $\bar{\nu }$ = 3,337, 3,302 (ν N–H), 3,087 (ν C–H), 1,642 (ν C=N), 1,547, 1,450 (ν C=C), 1,383, 1,280, 1,131 (ν SO_2_), 1,008 (δ C–H), 822, 777 (γ C–H), 581 (γ N–H) cm^−1^; ^1^H NMR (200 MHz): δ = 4.04 (s, 3H, OCH_3_), 7.08 (d, *J* = 5.1 Hz, 1H, 4-chloropyridine), 7.55 (s, 1H, pyrazine), 8.75–8.77 (m, 3H, 2H 4-chloropyridine and 1H NH + D_2_O exchangeable), 9.22 (s, 1H, pyrazine), 9.27 (br s, 1H, NH + D_2_O exchangeable) ppm; ^13^C NMR (50 MHz): δ = 54.64, 126.82, 135.66, 135.88, 140.01 (2C), 140.68, 141.96, 149.33, 154.49, 159.04 ppm; MS (−): *m*/*z* = 326 (15 %, [M−2H]^−^), 290 (100 %, [M−2H–Cl]^−^); MS (+): *m*/*z* = 341 (8 %, [M+2Na−H–NH_2_–O]^+^), 153 (100 %, [M+H−Cl–C_5_H_3_N–NH_2_–O]^+^).

#### *N′*-*(2*-*Chlorophenylsulfonyl)quinoline*-*2*-*carboximidamide* (**15**, C_16_H_12_ClN_3_O_2_S)

This compound was recrystallized from ethanol affording 0.97 g (70 %) **15**. M.p.: 161–162 °C; IR (KBr): $\bar{\nu }$ = 3,446, 3,331 (ν N–H), 1,640 (ν C=N), 1,616 (ν C=C), 1,527 (δ N–H), 1,277, 1,180, 1,106 (ν SO_2_), 1,030 (δ C–H), 801, 770, 628 (γ C–H), 575 (γ N–H) cm^−1^; ^1^H NMR (200 MHz): δ = 7.53–7.77 (m, 4H, 2H Ph and 2H quinoline), 7.88 (t, *J* = 7.0 Hz, 1H, quinoline), 8.06–8.23 (m, 4H, 2H Ph and 2H quinoline), 8.52 (d, *J* = 8.2 Hz, 1H, quinoline), 8.60 (br s, 1H, NH + D_2_O exchangeable), 9.30 (br s, 1H, NH + D_2_O exchangeable) ppm; ^13^C NMR (50 MHz): δ = 119.26, 127.79, 128.34, 129.02, 129.39, 129.58, 129.64, 131.12, 131.64, 132.03, 134.20, 138.52, 139.58, 146.33, 148.98, 159.57 ppm.

#### *N′-[(4-Chloropyridin-3-yl)sulfonyl]quinoline-2-carboximidamide* (**16**, C_15_H_11_ClN_4_O_2_S)

This compound was recrystallized from dioxane affording 1.1 g (80 %) **16**. M.p.: 347–350 °C (decomp.); IR (KBr): $\bar{\nu }$ = 3,380, 3,182 (ν N–H), 1,637 (ν C=N), 1,560 (ν C=C), 1,536 (δ N–H), 1,300, 1,148, 1,121 (ν SO_2_), 813, 771, 627 (γ C–H), 605, 592 (γ N–H) cm^−1^; ^1^H NMR (200 MHz): δ = 7.72–7.81 (m, 2H, 1H quinoline and 1H 4-chloropyridine), 7.91 (t, *J* = 7.0 Hz, 1H, quinoline), 8.09–8.19 (m, 3H, quinoline), 8.57 (d, *J* = 8.8 Hz, 1H, quinoline), 8.69 (br s, 1H, NH + D_2_O exchangeable), 8.77 (d, *J* = 5.4 Hz, 1H, 4-chloropyridine), 9.25 (s, 1H, 4-chloropyridine), 9.42 (br s, 1H, NH + D_2_O exchangeable) ppm; ^13^C NMR (50 MHz): δ = 119.36, 126.80, 128.38, 129.12, 129.44, 129.61, 131.19, 135.71, 138.61, 141.98, 146.35, 148.82, 149.40, 154.54, 159.90 ppm; MS (−): *m*/*z* = 713 (7 %, [2M+Na−3H]^−^), 613 (41 %, [2M+Na−2Cl−2NH_2_]^−^), 283 (61 %, [M+Na−3H−Cl−NH_2_−2O]^−^), 255 (100 %, [M+Na−2H–Cl–C_5_H_3_N]^−^); MS (+): *m*/*z* = 715 (25 %, [2M+Na−H]^+^), 353 (100 %, [M+Na−H–NH_2_]^+^), 153 (25 %, [M−H–NH_2_–Cl–C_5_H_3_N–SO_2_]^+^).

### X-ray crystallography

Good quality single-crystal specimens were selected for the X-ray diffraction experiments at *T* = 295(2) K. They were mounted with epoxy glue at the tip of glass capillaries. Diffraction data were collected on an Oxford Diffraction Gemini R ULTRA Ruby CCD diffractometer with CuKα radiation (*λ* = 1.54184 Å). The lattice parameters were obtained by least-squares fit to the optimized setting angles of the collected reflections by means of CrysAlis CCD [24]. Data were reduced by using CrysAlis RED [24] software by applying multi-scan absorption corrections (empirical absorption correction using spherical harmonics, implemented in the SCALE3 ABSPACK scaling algorithm). The structural resolution procedure was made using the SHELXS-97 package solving the structures by direct methods and carrying out refinements by full-matrix least-squares on *F* ^2^ using the SHELXL-97 program [25].

All H atoms bound with aromatic C atoms were placed geometrically and refined using a riding model with C–H = 0.93 Å and *U*_iso_(H) = 1.2 *U*_eq_(C). All H atoms bound with N atoms were placed geometrically and refined using a riding model with N–H = 0.86 Å and *U*_iso_(H) = 1.2 *U*_eq_(N). All interactions demonstrated were found by the PLATON program [26]. The following programs were used to prepare molecular graphics: ORTEPII [27], PLUTO-78 [28], and Mercury [29].

Full crystallographic details for compounds **3** and **4**, excluding structures features, have been deposited with the Cambridge Crystallographic Data Centre (deposition no. CCDC 869834 & 869835). These data may be obtained, on request, from the Director, CCDC, 12 Union Road, Cambridge, CB2 1EZ, UK (Tel.: +44-1223-336408; Fax: +44-1223-336033; E-mail: deposit@ccdc.cam.ac.uk or http://www.ccdc.cam.ac.uk).

### Tuberculostatic activity

The newly synthesized compounds were examined in vitro for their tuberculostatic activity against *M. tuberculosis* H_37_Rv strain and two “wild” strains isolated from tuberculosis patients: one (sp. 210) resistant to *p*-aminosalicylic acid (PAS), isonicotinic acid hydrazide (INH), etambutol (ETB), and rifampicine (RFP), and the another (sp. 192) fully sensitive to the administered tuberculostatics (Table 2). Investigations were performed by a classical test-tube method of successive dilution in Youmans’ modification of the Proskauer and Beck liquid medium containing 10 % of bovine serum [30, 31]. Bacterial suspensions were prepared from 14-day-old cultures of slowly growing strains and from 48-h-old cultures of saprophytic strains [32, 33]. Solutions of compounds in ethylene glycol were tested. Stock solutions contained 10 mg of compounds in 1 cm^3^. Dilutions (in geometric progression) were prepared in Youmans’ medium. Media containing no investigated substances and containing INH as reference drug were used for comparison. The incubation was performed at a temperature of 37 °C. The MIC values were determined as the minimum concentration inhibiting the growth of the tested tuberculous strains in relation to the probe with no tested compound.

### Antibacterial activity

Compounds **1**–**15** were dissolved immediately before use in 100 % DMSO at 5 mg/cm^3^ and further to 1 mg/cm^3^ in 10 % DMSO. Compounds were tested at serial dilutions in bacterial broth starting at 100 μg/cm^3^ (final concentration). *P. acnes* (ATCC 11827, lot 419697) was grown in thioglycollate nutrient broth (Hardy Diagnostics K29) for 72 h at 33 °C, then inoculated at the density equivalent to 0.5 McFarland standard and incubated with the test materials for another 72 h in an anaerobic environment. *B. linens* (ATCC 9174, lot 419862) culture was started from an agar plate, grown in nutrient broth (Hardy Diagnostics K243) for 24 h at 30 °C, then inoculated at the density equivalent to 1 McFarland standard and incubated with the test materials for another 24 h. At the end of the incubation the MIC was assessed optically as the lowest concentration of a test material which caused no bacteria growth [34]. This optical assessment was further confirmed by measuring the absorbance at 655 nm with the BioRad 3550-UV microplate reader. The MIC was defined as at least 50 % inhibition of the increase of OD.

### Cytotoxic activity

Passage for normal neonatal human dermal fibroblasts (ATCC PCS-201-010, lot 58243223) were grown in DMEM with 5 % calf serum (Hyclone). For the experiment, cells were plated in DMEM/5 % serum at 2,000 cells/well in 96 well plates (plate 598) and were exposed to test materials for 96 h. MAP and bFGF were used as the positive controls and water, 1, 0.5, and 0.25 % DMSO were used as the negative controls. Plate growth was stopped and cells were stained with a sulforhodamine B dye [35]. The dye was then dissolved and a colorimetric signal proportional to total cell/protein count was quantified with the BioRad microplate spectrophotometer 3550-UV at 570 nm with background subtraction at 660 nm and analyzed with Microplate Manager v.2 software for Macintosh (BioRad). Error bars represent standard errors of the mean (SEM). *P* values representing statistical significance were calculated using the *t* test.

### Antitumor activity

Compounds **2**–**16** were tested in the preliminary screening on a panel of 60 human tumor cell lines in the framework of the in vitro Development Therapeutic Program (DTP) at the National Cancer Institute (Bethesda, MD, USA). Cell lines were derived from nine different cancer types: leukemia, lung, colon, CNS, melanoma, ovarian, renal, prostate, and breast. Compounds were tested at one concentration (10 μM). Compound **15**, which passed the preliminary screening, was then tested at five different concentrations. Details of the system and the information which is encoded by the activity pattern over all cell lines have been published [36–38]. The antitumor activity of a test compound is given by the parameters for each cell line: *GI*_50_, i.e., the molar concentration of the compound that inhibits 50 % net cell growth, TGI, i.e., the molar concentration of compound leading to total growth inhibition, and *LC*_50_, i.e., the molar concentration of the compound leading to 50 % net cell death. Furthermore, a mean graph midpoint (MG_MID) is calculated for each of the mentioned parameters, giving an averaged activity parameter over all cell lines. For the calculation of the MG_MID, insensitive cell lines of the screen are included with the highest concentration tested. The selectivity of a compound with respect to one or more cell lines of the screen is characterized by the high deviation of the particular cell line parameter compared to the MG_MID value.
